# Developmental dynamics of cloned Mexican bighorn sheep embryos using morphological quality standards

**DOI:** 10.1002/vms3.242

**Published:** 2020-01-29

**Authors:** Sarahí Hernández Martínez, José E. Hernández Pichardo, José R. Vazquez Avendaño, Demetrio Alonso Ambríz García, María del Carmen Navarro Maldonado

**Affiliations:** ^1^ Department of Biology of Reproduction, Biological and Health Sciences Division Universidad Autónoma Metropolitana Iztapalapa Unit Iztapalapa Mexico; ^2^ Department of Agriculture and Animal Production, Biological and Health Sciences Division Universidad Autónoma Metropolitana Xochimilco Unit Mexico City Mexico

**Keywords:** bighorn, clones, embryo dynamics, morphological quality

## Abstract

The developmental dynamics of cloned Mexican bighorn sheep (*Ovis canadensis mexicana*) embryos were evaluated based on morphological quality standards. Categories determined by standards were correlated with the embryonic development stage, number of nuclei and viability. The results showed no differences in the blastocyst rate between the experimental (cloned Mexican bighorn sheep embryos) and control (parthenogenetic domestic sheep embryos) groups (*p* > .05), while type IV fragmentation was higher in clones (*p* < .05). The standards allowed for the identification of embryos that divided at least once or fragmented after 24 hr of culture. The highest percentage of morulae appeared at 96 hr, the final stages of development: nonsegmented, blocked, fragmented and blastocysts appeared at 192 hr. Embryonic quality decreased over time, making 96 hr the ideal time point to predict the final morphological quality of embryos. Nuclear staining of the morulae and blastocysts showed that higher embryo quality was associated with a higher percentage of normal and viable blastomeres. The evaluated criteria allowed for descriptions of the dynamics, stage and quality of cloned Mexican bighorn sheep embryos with a high degree of reliability. In addition, developmental anomalies, including fragmentation, multinucleation and blocking, were identified.

## Graphical abstract

Bighorn sheep cloned embryo development dynamics, Classification of cloned bighorn sheep embryos.

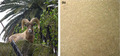

## 1 INTRODUCTION

Reproductive biotechnologies allow for efficient animal reproduction and the ex situ conservation of wild endangered species.

In Mexico, the bighorn sheep was originally distributed in the northern states of Nuevo Leon, Coahila, Chihuahua, Sonora, Baja California and Baja California Sur. Now it is restricted to three states: Mexican bighorn (*Ovis canadensis mexicana*) in north‐western Sonora and on Tiburon Island in the Sea of Cortez; Peninsular bighorn (*Ovis canadensis cremnobates*) in the northern two thirds of Baja California; and Weems’ bighorn (*Ovis canadensis weemsi*) in the southern third of Baja California Sur (Festa‐Bianchet, 2008).

Until 2008, the bighorn sheep was catalogued as least concern by the IUCN (Festa‐Bianchet, 2008). Yet, the International Convention for the Commerce of Flora and Wildlife Endangered Species (2015) has reported a reduction on populations of the Mexican species (2015).

Few has been done about the application of biotechnologies for assisted reproduction in this species, as ex situ strategies of conservation of species. Actual research on Mexican bighorn sheep (*O. c. mexicana*) is only directed to anatomic studies and population monitoring (Besser et al., 2013; Smith, Jenks, Grovenburg, & Klaver, 2014).

It is for this reason that the production of embryos by interspecies handmade cloning, offers an alternative for the conservation of endangered wild species. The application of reproductive biotechnologies on the endangered species implies to know the reaches of the technique, therefore, it is important that each step must be studied.

Studies of oocyte maturation, spermatic capacitation, fertilization and early embryonic development contribute to the progress of biotechnologies such as in vitro fertilization (IVF), intracytoplasmic sperm injection, somatic cell nuclear transfer (SCNT) and animal transgenesis (Ferré & Cattaneo, 2013). Thanks to SCNT, it is possible to produce individuals that are identical to their progenitor, and research has focused on the optimization of each step, with attention to in vitro embryonic development (IVED) (Ao, Liu, Cai, Wu, & Li, 2016), although the quality and viability of these embryos are lower relative to those produced in vivo. As a consequence, embryo evaluation by morphological classification is of great importance for the success of assisted reproduction techniques. This consists of selection of the best quality embryos in terms of internal and external morphological parameters, division rhythms and viability (Rocha et al., 2016). Such an approach represents an alternative method to increase IVED efficiencies by SCNT and in vitro embryo production for endangered wild and domestic species.

Early selection of in vitro‐produced embryos allows for increased embryo transfer rates, implantation and live offspring (Fabozzi et al., 2016). However, handmade cloning has not documented morphological evaluation or rates of embryo abnormalities such as fragmentation, multinucleation and development blocking because embryonic success is determined by the birth of live offspring.

The objective of the present study was to evaluate the developmental dynamics of cloned bighorn sheep (*O. c. mexicana*) embryos based on criteria proposed by The International Embryo Transfer Society (IETS). These criteria were used as the only existent resource for in vitro*‐*produced embryos in different species, to establish a reference for ontogenic development and possible alterations to which embryos are subjected, making early selection of the best quality embryos possible.

Interspecies cloning was performed using enucleated oocytes from domestic sheep (*Ovis aries*) as receivers of bighorn sheep (*O. c. mexicana*) somatic cells, because these species share the same number of chromosomes (2*n* = 54) (Delgadillo, Mejía, Berruecos, and Vásquez (2003).

## 2 MATERIALS AND METHODS

Reagents were obtained from Sigma‐Aldrich Chemical Co., unless otherwise indicated. Culture conditions consisted of 38°C, 5% CO_2_ and saturation humidity.

### 2.1 Animal management

Ovaries were collected from adult (age 3–4 years) domestic sheep (*O. aries*) at a local slaughterhouse. Skin tissue collection from a male adult (age of 5 years) bighorn sheep (*O. c. mexicana*) was performed at 5 hr *post mortem*.

### 2.2 Culture and cryopreservation of Mexican bighorn sheep fibroblasts

**Figure 1:**
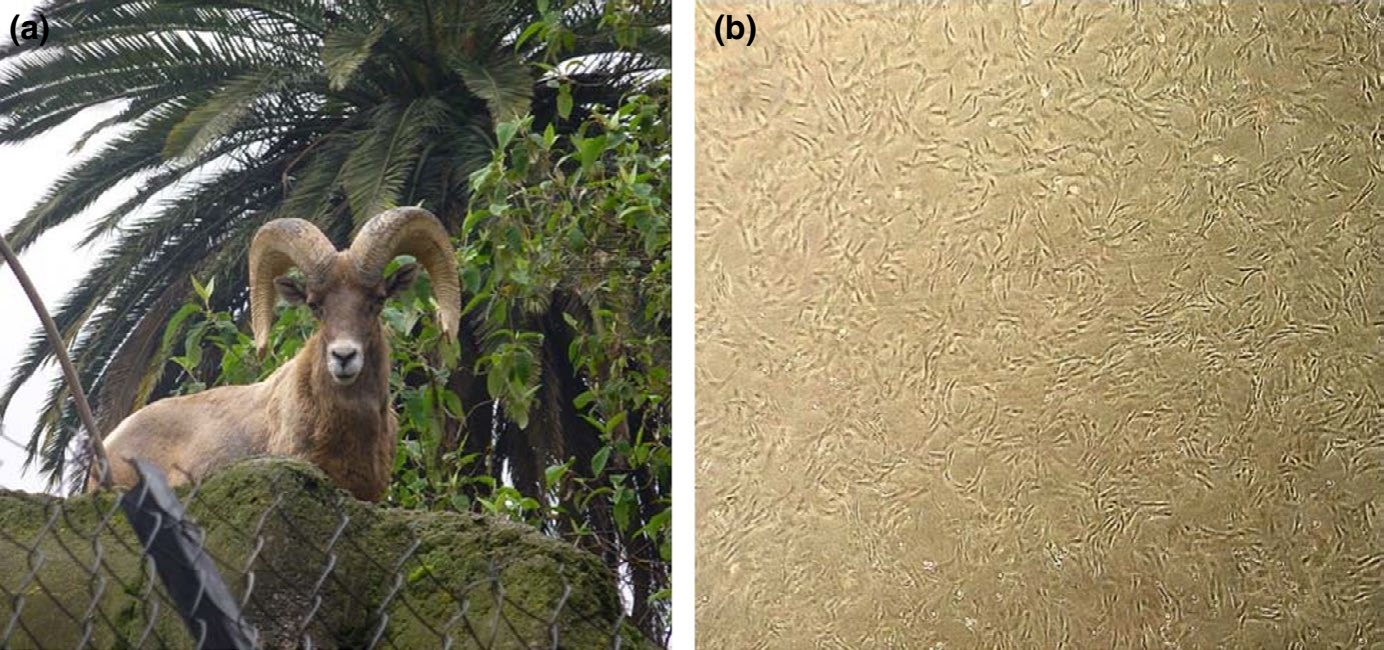
(a) Mexican bighorn sheep (*O. c. mexicana*), male, age 5 years. (b) Mexican bighorn sheep, male, age 5 years, post‐mortem ear skin fibroblasts, passage 5, 100% confluence, used as karyoplasts. 20× magnification

Cellular drift was made from 1 cm^2^ of the ear skin of a *post‐mortem* adult male bighorn sheep. The tissue was transported on ice to the laboratory, time lapsed was 5 hr. Once in the laboratory the tissue was cooled 24 hr before assay. After this period, the tissue was enzymatically disaggregated in collagenase type I and II (0.2%/0.2%, Gibco; in Dulbecco's phosphate‐buffered saline without calcium and magnesium, In Vitro S. A. CDMX, México). Cells in suspension were seeded into Dulbecco's Modified Eagle Medium (DMEM, In Vitro S. A.) supplemented with 10% fetal calf serum (FCS, Microlab, S. A. de C. V. CDMX, México) and 100× antibiotic‐antifungal (In Vitro S. A.). Every 7 days (d) for 4 weeks, cultures reaching 80%–100% confluence were used for the cellular passage. Fibroblasts were selected accordingly to Navarro‐Maldonado et al. (2015) procedure for evaluation of bighorn sheep epithelial cells morphology (Figure 1a and b). Once four passages were completed, cells (fibroblasts) were cryopreserved and stored at −80°C. Before cloning, fibroblasts were thawed and seeded. Fibroblasts at G0/G1 were synchronized by contact inhibition, leaving them in culture until they reached confluence (7 days); then, they were detached and maintained in TCM‐199 with Hepes (In Vitro S. A.) supplemented with 20% FCS (T20) (Vázquez et al., 2017) to use them as karyoplasts for interspecies handmade cloning.

### 2.3 In vitro maturation of oocytes

Ovaries were transported to the laboratory (time lapsed 2 hr) in physiological solution (0.9% NaCl and 1% antibiotic‐antifungal) at 30–35°C. Oocyte cumulus complexes (OCC) were aspirated from the follicles (2–5 mm in diameter) in TCM‐199 with Hepes supplemented with 100 UI/ml sodium salt heparin. The recovered OCC were classified according to their morphology (ASEBIR, 2015) and number of granulosa cell layers. Those selected were incubated for 21 hr in in vitro maturation (IVM) medium (TCM‐199 supplemented with cysteine [0.57 mM], D‐glucose [3.05 mM], polivinilic alcohol [PVA] [0.1%], sodium pyruvate [0.91 mM], 10% FCS, hCG [5 UI/ml, Ferring Pharmaceuticals], rFSH [0.1 UI/ml, Merck Serono, Darmstadt, Germany], antibiotic‐antifungal [2%] and epidermal growth factor [EGF, 10 ng/ml]). The criteria for IVM evaluation were cumulus cell expansion and presence of the first polar body (1PB) (Vázquez et al., 2017). Then, oocytes were divided into two groups: experimental, cloned bighorn sheep embryos (EG), and control, parthenogenetic domestic sheep embryos (CG).

### 2.4 Production of cloned embryos

Selected EG oocytes were first incubated for 1 hr in demecolcine (0.5 µg/ml) for extrusion of the metaphase plate, and then incubated in pronase (2 mg/ml) to digest the pellucida zone (PZ). Finally, they were enucleated by bisectioning with a microblade.

The enucleated oocytes (cytoplasts) were immersed in phytohaemagglutinin (0.5 mg/ml) for approximately 4 s, after that, a single somatic cell (karyoplast) was placed between two cytoplasts by forming the cellular triplets.

For the fusion of the triplets, a cell fusion machine (Instrument BLS) connected to a fusion chamber (BTX microslide, model 450) was used, covering the cells with fusion media containing d‐mannitol (0.3 M) and PVA (1 mg/ml). The cellular triplets (cytoplast–karyoplast–cytoplast) were fused using an alternating current (AC) of 9 V to apply a pulse of 100 V/mm of direct current (DC) for 9 µsec. Mexican bighorn sheep cloned embryos were incubated for 3 hr in embryo development culture for karyoplast reprogramming (Vajta et al., 2003).

### 2.5 Production of parthenogenetic embryos

As handmade cloning needs chemical activation of zygotes produced, based on studies by Jena et al. (2012) in order to have a control group with a similar chemical activation, that is, the artificial activation of a diploid nucleus (2n), we produced parthenogenetic *O. aries* embryos.

Similarly to oocytes used as cytoplasts for embryo cloning which lack of pellucid zone, oocytes for CG were zona free. Thus, they were also incubated in the WOW system and cleavage medium (Cook Medical) until the activation process was performed.

### 2.6 Activation and embryo culture

In both groups, embryos were incubated in calcium ionophore A23187 (8 µM/ml) for 5 min and in 2 mM 6‐dimethylaminopurine for 5 hr. Then, between 10 and 20 embryos from EG and CG treatments were cultured in a WOW system for 8 days (192 hr) (Vajta et al., 2003). Embryo culture was initiated in drops of 450 µl of cleavage medium for the first 96 hr and in blastocyst medium for the last 96 hr (Cook Medical).

### 2.7 Assessment of embryo quality

Criteria evaluated to determine the embryo quality were based on the IETS Manual (Stringfellow & Givens, 2011):

Segmentation rate: Number of embryos with at least one cell division.

Development stage: Number of embryos per development stage evaluated at 24, 96 and 192 hr of culture, plus fragmented stages.

Embryo morphology: Evaluated at 24, 96 and 192 hr of culture, including own modifications for IVED of cloned embryos. Quality categories were as follows:

1 «Excellent or good»: Spherical embryonic mass, uniform in colour and symmetry, intact and viable at 85%.
2 «Fair»: Moderate irregularities in size, symmetry and colour, with least 50% intact, and a viable mass.
3 «Poor»: Irregularities important in size, symmetry and colour, with least 25% intact, and a viable mass.
4 «Dead or degenerating»: Degenerating embryos or 1‐cell embryos, nonviable.

Fragmentation: Embryos showing more than eight segmentations or >50% of cytoplasm fragmented (type IV fragmentation) at 24–192 hr (Stone, Greene, Vargyas, Ringler, and Marrs 2005), nonviable.

Viability and nuclei counts: In morulae and blastocysts, the percentages of blastomere and viable embryos were determined (>75% live blastomeres). Normal blastomere (nucleus:blastomere, 1:1), fragmentation (blastomere without nuclei) and multinucleation (blastomere with more than one nucleus) rates were determined. Double Hoechst 33342 (5 µg/ml) and propidium iodide (300 µg/ml) staining was performed to evaluate the embryos under an epifluorescence microscope (Nikon Eclipse E600). To observe the nuclei stained in blue (Hoechst 33342), a UV‐2A (wavelength: 330–380 nm) filter was used. For damaged or dead cells stained in red (propidium iodide), a G‐2A (wavelength: 510–560 nm) filter was used (Rodríguez, Romo, Ducolomb, Casas, & Hernández, 2017).

### 2.8 Statistics

A chi‐squared (*χ* ^2^) test was applied to compare groups with a confidence level of .05. To correlate morphology criteria with viability and normal, fragmented and multinucleated blastomeres, R Pearson's test was applied with the same confidence level. We used the statistical package NCSS version 2007.

## 3 RESULTS

From 567 abattoir obtained sheep ovaries, 2374 OCC were collected. Of these, 798 were selected for IVM, 697 matured oocytes were selected for zona removal. Of these 456 were selected for enucleation, which resulted into 392 survived enucleated oocytes. These 392 cytoplasts were attached to Mexican bighorn sheep cells. Finally, 175 interspecies constructs were activated and cultured for up to 7 days in vitro.

### 3.1 Embryo production: development and segmentation rate

**Figure 2:**
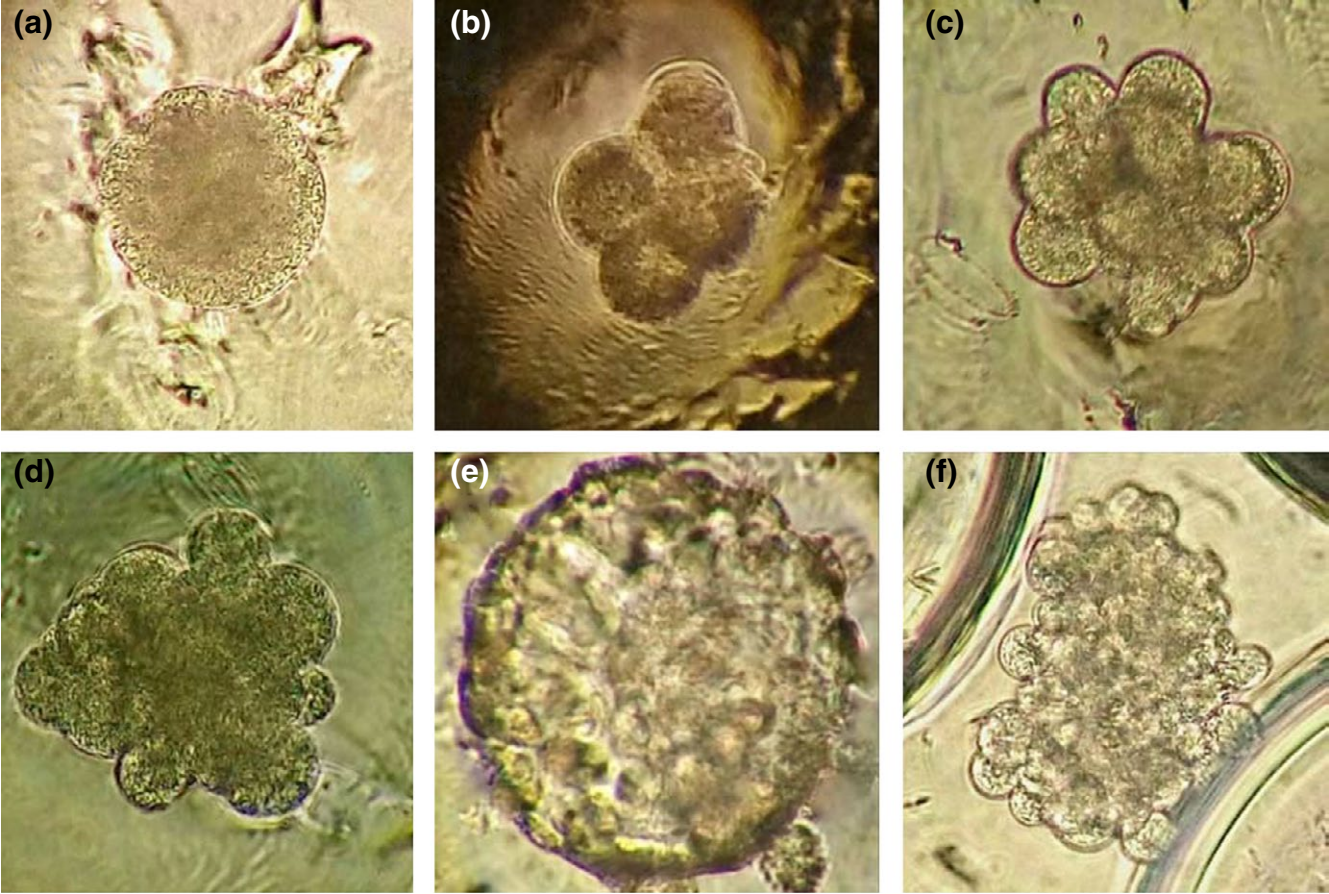
Development stages of Mexican bighorn sheep (*O. c. mexicana*) cloned embryo. (a) Zygote (96 hr culture). (b) 2‐to 4‐cell (24 hr culture). (c) Morula (96 hr culture). (d) Compact morula (96 hr culture). (e) Blastocyst (192 hr culture). (f) Fragmented (24 hr culture). 40× magnification

Table 1 shows no significant differences (*p* > .05) between groups for segmented embryos (91.4% EG vs. 83.6% CG) and blastocysts (14.3% EG vs. 19.7% CG). In both groups, most embryos reached at least one cell division within the first 24 hr of culture, and a greater proportion of morulae developed at 96 hr, while blastocysts appeared at 192 hr (Figure 2a–e). At this time, the inhibition of embryonic development was detected at the 2‐ to 8‐cell and morula stages. Embryo type IV fragmentation was detected following 24 hr of culture and showed significant differences between the three evaluated culture time points (*p* < .05) (Figure 2f, Table 2).

**Table 1:** Segmentation potential and blastocyst formation of cloned and parthenogenetic embryos. Each value represents mean ± *SEM*

Group	Total	Embryos *n* (%)	
		Segmented	Blastocysts
EG	175	160 (91.4)^a^	25 (14.3)^a^
$\bar{x}$±*SEM*	(17.5 ± 0.4)	(16.0 ± 0.3)	(2.5 ± 0.3)
CG	122	102 (83.6)^a^	24 (19.7)^a^
$\bar{x}$±*SEM*	(12.2 ± 0.6)	(10.2 ± 0.6)	(2.4 ± 0.3)

Note

Abbreviations: CG, Control group. *O. aries* parthenogenetic embryos; EG, Experimental group. *O. c. mexicana*‐cloned embryos.

a Similar superscripts in the columns indicate no significant differences between segmented embryos and blastocysts among EG and CG (*p* > .05).

**Table 2:** Embryonic development stage evaluated at 24, 96 and 192 hr of culture. Each value represents mean ± *SEM*

Stages†	24 hr		96 hr		192 hr	
	*n* (%)		*n* (%)		*n* (%)	
	EG	CG	EG	CG	EG	CG
1 cell	55 (31.5)	35 (28.7)	15 (8.6)	20 (16.4)	15 (8.6)	20 (16.4)
$\bar{x}$±*SEM*	5.5 ± 0.5	3.5 ± 0.5	1.5 ± 0.3	2.1 ± 0.5	1.5 ± 0.3	2.0 ± 0.5
2–8 cells	107 (61.1)	86 (70.5)	51 (29.1)	42 (34.4)	39 (22.3)	27 (22.1)
$\bar{x}$±*SEM*	10.7 ± 0.6	8.6 ± 0.5	5.1 ± 0.4	4.2 ± 0.5	3.9 ± 0.4	2.7 ± 0.4
Morulae	0	0	58 (33.2)	42 (34.4)	44 (25.1)	29 (23.8)
$\bar{x}$±*SEM*	0 ± 0	0 ± 0	5.8 ± 0.4	4.2 ± 0.5	4.4 ± 0.2	2.9 ± 0.3
Blastocysts	0	0	0	0	25 (14.3)	24 (19.7)
$\bar{x}$±*SEM*	0 ± 0	0 ± 0	0 ± 0	0 ± 0	2.5 ± 0.2	2.4 ± 0.2
Fragmented	13 (7.4)^a^	1 (0.8)^b^	51 (29.1)^a^	18 (14.8)^b^	52 (29.7)^a^	22 (18.0)^b^
$\bar{x}$±*SEM*	1.3 ± 0.7	0.1 ± 0.3	2.9 ± 0.4	1.8 ± 0.5	5.2 ± 0.3	2.2 ± 0.5

Note

Abbreviations: CG, Control group. *O. aries* parthenogenetic embryos. (*n* = 122); EG, Experimental group. *O. canadensis mexicana*‐cloned embryos. (*n* = 175).

Most embryos reached one cell division at 24 hr culture. Morulae developed at 96 hr and blastocysts at 192 hr. Embryo development blocking appeared in 2‐ to 8‐cells and morula, in both groups. Embryo fragmentation type IV appeared at 24 hr, showing significant statistical differences between the three culture times evaluated.

† Embryo stage codes are grouped to unify values in such a way that morula stages included codes for early (3) and compacted morulae (4), while blastocyst stages included codes for early (5), late (6) and expanded blastocysts (7), in agreement with the IETS Manual.

‡ Annexed stage: Involves type IV fragmentation proposed by Stone et al. (2005).

a,b Different superscripts between the columns show significant differences between groups and hr (*p* < .05).

### 3.2 Embryo morphology

**Figure 3:**
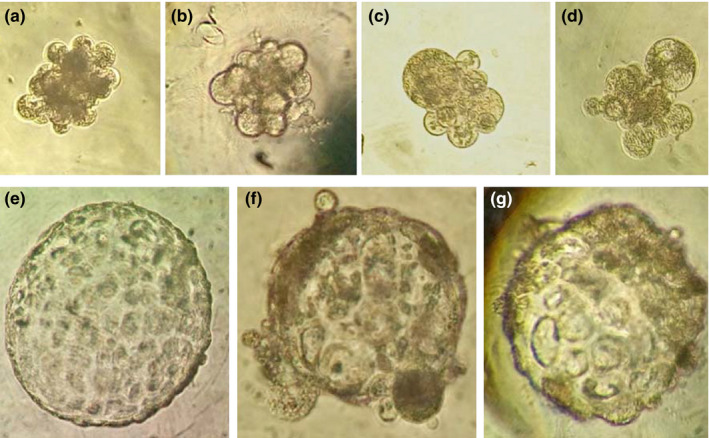
Quality categories of Mexican bighorn sheep (*O. c. mexicana*) cloned embryo. (a) Morula quality 1 «Excellent or good». (b) Morula quality 2 «Fair». (c) Morula quality 3 «Poor». (d) Morula quality 4 «Dead or degenerated». (e) Blastocyst quality 1 «Excellent or good». (f) Blastocyst quality 2 «Fair». (g) Blastocyst quality 3 «Poor». 40× magnification

For IVED, morphological quality was negatively correlated with the culture period in both groups. Although there was a high percentage of embryos in quality categories 1 «Excellent or good» and 2 «Fair» at 24 hr of culture, this time point was not the most suitable to make a successful evaluation because culture quality category 1 diminished considerably (*p* < .05) from 96 hr onward, and quality categories 3 «Poor» and 4 «Dead or degenerating» rose (*p* < .05). From 96 to 192 hr of culture, embryo quality remained constant (*p* > .05), and morulae developed. These periods and developmental stages can be considered suitable to predict the embryo morphology quality with a high degree of reliability (Figure 3a–g, Table 3).

**Table 3:** Embryo morphological quality evaluated at 24, 96 and 192 hr of culture. Each value represents mean ± *SEM*

Morphological Quality^ ^IETS	24 hr		96 hr		192 hr	
	*n* (%)		*n* (%)		*n* (%)	
	EG	CG	EG	CG	EG	CG
1. Excellent or good	122 (69.7)^A^	72 (59.0)^A^	3 (1.7)^Ab^	11 (9.0)^bB^	11 (6.3)	12 (9.8)
$\bar{x}$±*SEM*	12.2 ± 0.4	7.2 ± 0.6	0.3 ± 0.3	1.1 ± 0.4	1.1 ± 0.4	1.2 ± 0.5
2. Fair	52 (29.7)	41 (33.7)	54 (30.9)	41 (33.7)	45 (25.7)	32 (26.2)
$\bar{x}$±*SEM*	5.2 ± 0.3	4.1 ± 0.4	5.4 ± 0.4	4.1 ± 0.4	4.5 ± 0.4	3.2 ± 0.2
3. Poorx ® ± *SEM*	0^ A^	2 (1.6)^A^	85 (48.5)^aB^	43 (35.2)^bB^	68 (38.9)	50 (41.0)
$\bar{x}$±*SEM*	0 ± 0	12.2 ± 3.0	8.5 ± 0.5	2.7 ± 0.3	6.8 ± 0.6	5 ± 0.7
4. Dead or degenerating	1 (0.6)^A^	7 (5.7)^A^	33 (18.9)^ B^	27 (22.1)^B^	51 (29.1)	28 (23.0)
$\bar{x}$±*SEM*	0.1 ± 0.3	0.7 ± 0.8	3.3 ± 0.6	2.7 ± 0.4	5.1 ± 0.7	2.8 ± 0.4

Note

Abbreviations: CG, Control group. *O. aries* parthenogenetic embryos. (*n* = 122); EG, Experimental group. *O. canadensis mexicana*‐cloned embryos. (*n* = 175).

Morphological quality negatively correlated with culture period for EG and CG. A high percentage of embryos were quality 1 «Excellent or good» and 2 «Fair» at 24 hr culture. At 96 hr quality 1 decreased as quality 3 «Poor» and 4 «Dead or degenerating» raised (*p* < .05). From 96 to 192 hr culture, embryo qualities remained constant and morula developed, making these times and development stage suitable to predict embryo morphology quality.

a,b Different superscripts in the columns indicate significant differences between groups during the three evaluated culture periods.

A,B Different superscripts in the columns indicate significant differences between the time points of each group (*p* < .05), taking into consideration the 24 to 96 hr and 96 to 192 hr culture periods.

### 3.3 Morula viability

Blastomere viability showed a positive correlation with morphological quality (EG *r* = 0.937 and CG *r* = 0.869). There was a greater percentage of viable blastomeres as embryo quality improved (Table 4).

**Table 4:** Blastomere condition in the morula stage (96 hr). Each value represents mean ± *SEM*

Group	Morphological quality IETS	Blastomere (%)				
		Mean ± *SEM*	Viable	‡Normal	Multinucleated	Fragmented
EG	1	11.8 ± 0.2	95.4%	79.7%	15.2%	3.3%
	2	20 ± 0.1	76.9%	55.9%	3.5%	41.5%
	3	10 ± 0.2	59.3%^a^	33.3%^a^	0%	67.0%^a^
	4	13 ± 0.4	15.0%^a^	16.3%^a^	3.8%	80.7%^a^
	*r*		0.937	0.845	0.434	−0.904
CG	1	14.5 ± 0.7	96.5%	82.1%	3.4%	28.9%
	2	13.8 ± 0.5	82.5%	68.9%	15.9%	14.4%
	3	13.5 ± 0.6	33.3%^b^	19.6%^b^	7.4%	40.7%^b^
	4	14 ± 0	0%^b^	0%^b^	0%	78.6%^b^
	*r*		0.869	0.778	0.091	−0.458

Note

*r* = Pearson correlation with a confidence level of .05 applied for blastomere viability, showed a positive correlation with morphological quality and with nuclei counting, indicating a greater percentage of viable blastomeres and the highest normal blastomere percentage as morula quality improves.

Abbreviations: CG, Control group. *O. aries* parthenogenetic embryos. (*n* = 15); EG, Experimental group. *O. c. mexicana* cloned embryos. (*n* = 13).

‡ Nucleus‐blastomere relationship (1:1).

a,b Different superscripts in the columns indicate significant differences between groups (*p* < .05).

### 3.4 Numbers of nuclei in the morula stage (96 hr)

**Figure 4:**
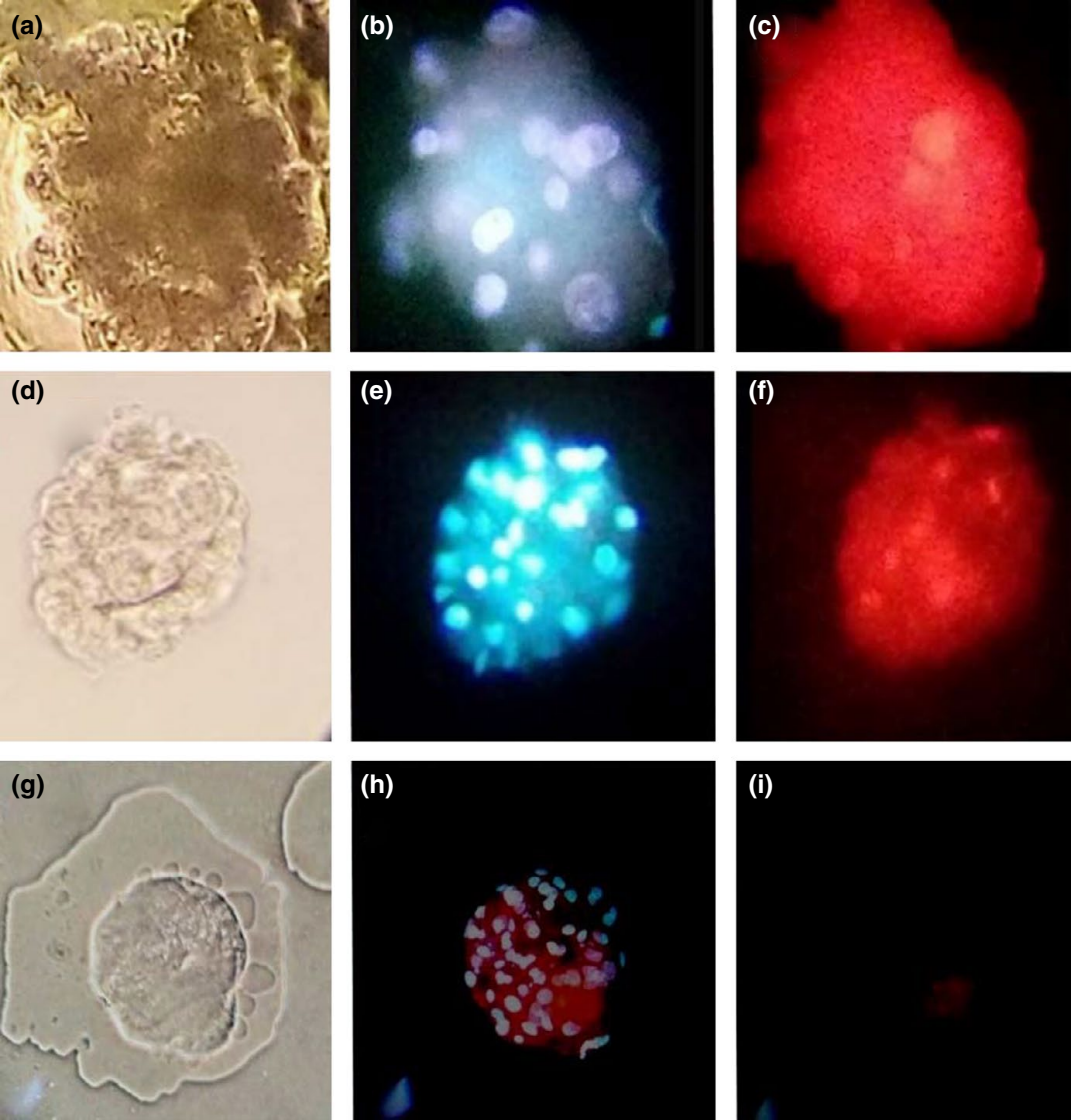
Nuclei and vital staining of Mexican bighorn sheep (*O. c. mexicana*) cloned embryos. (a) Morula in clear field. (b) Hoechst stained morula. (c) Propidium iodide stained Morula. 40× magnification. (d) Blastocyst clear field. (e) Hoechst stained blastocyst. (f) Propidium iodide stained Blastocyst. (g) Zona free parthenogenetic *O. aries blastocyst*, Clear field. (h) Hoechst stained. (i) Propidium iodide stained. 20× magnification

There was a positive correlation between the number of nuclei and the normal blastomere rate, indicating that the better the morphological quality is, the higher the normal blastomere percentage. With the exception of quality category 3 in EG and quality category 4 in CG, all other quality categories presented multinucleated blastomeres, with the highest percentage for quality category 1 in EG (15.2%) and quality category 2 in CG (15.9%) (both groups presented a positive correlation). Blastomere fragmentation was present in all quality categories, with a minimum percentage for quality category 1 in EG (3.3%) and quality category 2 in CG (14.4%). This variable negatively correlated with morphological quality because blastomere fragmentation diminished as embryo quality improved (Figure 4a–c, Table 4).

### 3.5 Blastocyst blastomere viability (192 hr)

Better morphological embryo quality resulted in a greater increase in the viable blastomere proportion (EG: *r* = 0.913 and CG: *r* = 0.945) (Figure 4d–i, Table 5).

**Table 5:** Viability in the blastocyst stage (192 hr). Each value represents mean ± *SEM*

Group	Morphological quality IETS	Mean ± *SEM*	Viable blastomeres (%)
EG	1	43.4 ± 3.7	95.9^a^
	2	47.8 ± 5.7	85.6^a^
	3	52.0 ± 0.8	70.6^a^
			*r* = 0.913
CG	1	41.5 ± 2.2	95.4^a^
	2	42.5 ± 5.8	85.8^a^
	3	22.9 ± 2.0	73.9^a^
			*r* = 0.945

Note

*r* = Pearson correlation with a confidence level of .05, related to morphological quality showed that, the better morphological embryo quality, the viable blastomere proportion increases.

Abbreviations: CG, Control group. *O. aries* parthenogenetic embryos. (*n* = 14); EG, Experimental group. *O. c. mexicana* cloned embryos. (*n* = 12).

a Similar superscripts in the columns indicate no significant differences between groups (*p* < .05).

Nucleus counting indicated an average of 46.7 ± 8.1 for EG blastocysts and 44.2 ± 9.9 for CG blastocysts (*p* > .05).

## 4 DISCUSSION

In this study, the segmentation rate in EG embryos (91.4%) and CG embryos (83.6%) was superior to that reported in caprine (74%) (Jena et al., 2012). Also, EG segmentation rate was superior to that reported in ovine (66.9%) (Khan et al., 2018) handmade cloned embryos. In cloned bovine embryos, the segmentation rate is affected by the activation method, and the time lapse between fusion and activation (Akagi, Matsukawa, & Takahashi, 2014), which may explain why the results in the present study differ from those of previous authors. Khan et al. (2014) reported a 92.5% segmentation rate in caprine cloned embryos cultured in G1/G2 media. In this study, human sequential media were used, which could have similarly influenced the segmentation rate.

It is known that sequential media tend to generate more successful developmental rates, because they consider the different metabolic and morphological requirements for each stage of embryo development (Haydar, Turan, Cihan, Bilgen, & Mustafa, 2012).

The developmental dynamics of cloned embryos are very particular but have not been well studied relative to those of IVF‐produced embryos. Based on IETS criteria, during the first 24 hr of culture, major segmentation occurs (2‐ to 8‐cell stages) (rates: EG 61.1% and CG 70.5%). This is desirable, as it was documented in buffalo embryos that those dividing earlier (54%) have major potential to develop into better quality blastocysts with a lower apoptotic index (Kaith et al., 2015). At 96 hr, morulae appeared, which differs from the study by Shabankareh and Zandi (2010), in which morulae appeared 6 days after IVF of ovine embryos, indicating that cloned embryos are advanced by up to 48 hr.

Blastocysts rate in this work was different to the reported for two other wild sheep species. Hajian et al. (2011) produced Esfahan mouflon cloned embryos (7.6%), these results are lower than those found in this work (14.3%), but are similar to the one reported by Pan, Zhang, Guo, and Wang (2014). They produced 15.7% blastocysts of Argali (*Ovis ammon*) adult (age 3–4 years) sheep, by traditional interspecies nuclear transfer.

Blastocysts appeared at 192 hr of culture (EG 14.3% and CG 19.7%) (*p* > .05), similarly to handmade cloned bighorn sheep female blastocysts (15.0%) produced by Vázquez et al. (2017). It is known that specific characteristics of fibroblasts such as sex (Sandhu et al., 2016), age (Tian, Kubota, Enright, & Xiangzhong, 2003) and passage number (Zhang et al., 2009) determine the development success rate. These variables act directly on the nuclear reprogramming and embryo genome activation. The presence of blastocysts at 192 hr is in agreement with the results of Shabankareh and Zandi (2010) for ovine IVF embryos. At the beginning of culture, cloned embryo segmentations initiate quickly and then become slower entering the blastocyst stage.

At 192 hr of culture, we identified the development blocking. This depends on species, oocyte origin and quality, type and composition of culture media and the activator agent (Kouamo & Kharche, 2015).

It is explained by the activation failure of genes related to development and others failures to produce apoptosis (Greenwood & Gautier, 2005), or by incorrect embryo genome activation (8‐to16‐cell stage) (García, Marinho, Lunardelli, Seneda, & Meirelles, 2015). These mechanisms can explain why 2‐ to 8‐cell or morula embryos were unable to continue developing.

Accordingly to Lagutina, Fulka, Lazzari, and Galli (2013), this activation failure could be due to the species origin. They say that, as species are phylogenetically distanced, that is chimpanzee embryos produced by interspecies cloning using chimpanzee fibroblasts fused with the enucleated bovine oocytes, activation failure will be more evident than when species are close phylogenetically. In this work, the interspecies cloning was performed with two species that are phylogenetically close (Mexican bighorn sheep/domestic sheep), since they share the same number of chromosomes 2*n* = 54 (Delgadillo et al., 2003).

Palma et al. (2012) showed that reproduction between phylogenetically close species (*O. canadensis* and *O. aries* hybrids) allows to obtain a similar in vitro embryonic division rate and developmental dynamics between groups. This was reinforced by the sequencing of Interferon *tau* (protein produced by the embryonic trophoctoctoderm during the critical period of maternal recognition of pregnancy in ruminants), which allowed to determine that the association of its amino acids between bighorn sheep and Pelibuey hybrids, show a greater identity with the members of the *Ovis* gender to which they belong, than with other species.

Monitoring embryonic development allowed the identification of type IV fragmentation starting at 24 hr of culture (Figure 2f), with significant differences between groups during the three culture periods (*p* < .05). Type IV fragmentation has not been described before during handmade cloning. In conventional cloning, this anomaly occurs in 17.7%–52.4% of sheep embryos and is linked to chromosomal disorders, defects in parental genotype, the instability of actin microfilaments (Xue et al., 2011) and cell fusion that destabilizes the double lipid layer in the cell membrane (Im et al., 2005). Moreover, handmade cloned embryos are produced from the fusion of three cells (two cytoplasts and one karyoplast), which dynamics and coordination between microtubules and microfilaments during fusion as well as cytokinesis, can fail leading to embryo fragmentation (Alikani, Schimmel, & Willadsen, 2005). Fragmentation was also observed in CG embryos, confirming its diverse origin.

In both groups, morphological quality diminished as the embryo culture period elapsed. Some embryos classified at 24 hr of culture as quality category 1 suffered alterations (cytoplasmic fragmentation or development blocking) upon the completion of 192 hr of culture, increasing the percentage of embryos in quality categories 3 and 4. However, from 96 to 192 hr, embryos of quality categories 1, 2 and 3 remained stable. For this reason, the early evaluation of embryo quality at 96 hr of culture is considered the most appropriate period to predict final embryo quality. Considering that morulae are present at 96 hr of culture, embryos with the potential to reach the blastocyst stage can be predicted and selected for transfer.

Although there are alternative systems for the evaluation of embryos, none of them have replaced the simplicity and accessibility of morphological evaluation using clear field microscopy. Thus far, there have been no studies of the morphological quality of cloned sheep embryos. Hence, this study is important for the implementation of morphological quality evaluations in wild sheep embryos, based on IETS criteria, which are internationally known and successfully applied.

Morula morphological quality positively correlated with viability, which is consistent with findings from IVF ovine morulae (Rodríguez et al., 2017). Morulae showed lower fragmentation or multinucleation, suggesting that cytoplasm portions produced by embryo fragmentation impact viability, which is accentuated as culture time elapses, compromising embryonic developmental potential.

Multinucleation has been well studied in human IVF embryos, not so in other species. It occurs during the first (57.1%) and second segmentations (50.0%) due to cytokinesis failure, provoking an arrest in cytoplasm division and resulting in the presence of more than one nucleus per blastomere (Van Royen et al., 2003). For human embryos in quality category 1, the multinucleation rate is 5%–7%, while for those in quality category 3, the percentage rises to 25% (Desai et al., 2014). In the present study, the multinucleation rate was 3.5% for cloned embryos in quality category 2 (EG) and 3.4% for CG embryos in quality category 1.

Fragmentation is present at a high frequency during the first embryo segmentation (>40%) and is associated with chromosomic anomalies, blastomere multinucleation and chromosomic mosaicism, processes that reduce the embryonic potential (Halvaei et al., 2016). della Ragione et al. (2007) predicted the implantation success of blastocysts by determining the morula fragmentation rate; those that showed ≤10% cytoplasm fragmentation were more successful than those that showed >10%–50% fragmentation. The latter embryos showed chromosomal alterations related to distortion in the planes of division, which causes poor compaction and embryo cavitation, resulting in the formation of abnormal blastocysts. Stigliani, Anserini, Venturini, and Scaruffi (2013) noted that the greatest embryonic fragmentation occurred with lower volume; meaning fewer available mitochondria, depriving the embryos of important DNA for progression in development. Considering the optimal level of fragmentation described by della Ragione et al. (2007), this work showed that the transferable embryo quality for morulae should be quality category 1 for EG embryos (3.3%).

In terms of viability and quality, blastocysts showed the same pattern as morulae. This criterion has not been described previously for handmade cloned embryos.

Regarding the number of nuclei in cloned embryos observed here, there were 46.7 ± 8.1 nuclei for EG and 44.2 ± 9.9 for CG (*p* < .05). Hosseini et al. (2015) recorded 125 ± 11.1 nuclei for handmade cloned caprine embryos, while those produced by conventional cloning showed 122 ± 10.5 nuclei. Gómez, Ramírez, and Ruíz (2017) reported 77.5 ± 8.2 nuclei for cloned bovine blastocysts and 78.1 ± 7.6 nuclei for parthenogenetic embryos. These numbers are higher than those in the present study. Cao et al. (2011) obtained 39 ± 3 nuclei in blastocysts produced by conventional cloning and 39 ± 17 nuclei in parthenogenetic swine embryos, which are lower numbers than those in the present study.

Gómez et al. (2017) indicated that variability in blastomere numbers is attributable to the species, embryo production technique and embryo manipulation. In handmade cloning, cytoplasts are subjected to major manipulation, i.e. manual enucleation, which is harmful to embryonic development and directly influences the cytokinesis, since it is known that the first embryo division is guided by the cytoplasmic transcripts of oocytes. In this way, the number of nuclei can be related to the fragmentation of the cytoplasm, since embryo volume is reduced and consequently forms a smaller blastocyst with few number of cells and nuclei.

## 5 CONCLUSION

Embryo morphological evaluation criteria (IETS) allowed for evaluation of the dynamics, stage and quality of cloned bighorn sheep embryos and anomalous development processes (blocking, fragmentation, multinucleation) with a high degree of reliability. In addition, at 96 hr of culture, it was possible to determine the potential development of morulae into better quality blastocysts. The production of 14.3% cloned blastocysts from a *post‐mortem O. c. mexicana* adult male allowed us to propose handmade cloning as an in vitro ex situ conservation strategy for wild endemic species at risk.

## CONFLICTS OF INTEREST

The authors declare no conflicts of interest to disclose.

## AUTHOR CONTRIBUTIONS

Sarahí Hernández Martínez and José Roberto Vazquez Avendaño performed the experiments; José Ernesto Hernández Pichardo y María del Carmen Navarro Maldonado drafted the manuscript, Demetrio Alonso Ambríz García assisted in laboratory work and analysed the data, María del Carmen Navarro Maldonado designed the study.
